# Supplementary material for: Teenage pregnancy and timing of first marriage in Cameroon—What has changed over the last three decades, and what are the implications?
Source: PLoS One. 2022 Nov 17;17(11):e0271153. doi: 10.1371/journal.pone.0271153 (PMC9671313; doi:10.1371/journal.pone.0271153)
Supplement: Attachment — Submitted filename: PLOS TeenPreg Cameroon Rebuttal Letter.docx [file pone.0271153.s004.docx]

September 7, 2022

The Editor,

*PLOS ONE Journal*

**RE: Rebuttal Letter**

Dear Editor,

We thank you for the comments on our manuscript. We present below our response to each of the points raised by the academic editor and reviewers.

A. **Comments by the Editor**
1. Please ensure that your manuscript meets PLOS ONE's style requirements, including those for file naming. The PLOS ONE style templates.

**Response**: We have followed the formatting requirements and revised accordingly.

2. In your Data Availability statement, you have not specified where the minimal data set underlying the results described in your manuscript can be found. PLOS defines a study's minimal data set as the underlying data used to reach the conclusions drawn in the manuscript and any additional data required to replicate the reported study findings in their entirety. All PLOS journals require that the minimal data set be made fully available. For more information about our data policy, please see <http://journals.plos.org/plosone/s/data-availability>.

**Response**: We are uploading the study’s minimal underlying data set as Supporting Information files in Excel format (S1 Fig, S2 Fig, S3 Fig).

3. We note that you have stated that you will provide repository information for your data at acceptance. Should your manuscript be accepted for publication, we will hold it until you provide the relevant accession numbers or DOIs necessary to access your data. If you wish to make changes to your Data Availability statement, please describe these changes in your cover letter and we will update your Data Availability statement to reflect the information you provide.

**Response**: Please see our response on Item #2 above (we have now provided supporting information files instead.

4. Please review your reference list to ensure that it is complete and correct.

**Response**: We have reviewed the list and all 40 references listed have been cited at least once in the manuscript.

B. **Comments by Reviewer #1**
1. The introduction lacks a strong motivation for the study. The paragraph placed from line 79 would have been best put as the last paragraph after a strong case has been made for the study. Lines 85 to 100 seem at best misplaced and at worst unnecessary.

**Response**

- The first paragraph of the introduction (Lines 49-54) was designed to provide a strong motivation for the study. We have expanded on the consequences of teenage pregnancy and motherhood.
- We have moved up the text in Lines 85-100, making the paragraph from line 79, the last paragraph of the introduction.

2. The Methods section in both the abstract and the manuscript need more detail, readers need to be clear about study population, inclusion and exclusion criteria if any, study setting, data collection (clearly state that secondary data analysis was done on data collected for DHS during the period ..... through .....), variables, data analysis etc. Descriptions of how variables were named, coded and recoded that the authors put in the abstract were misplaced, these are better described under variables in the methods section.

**Response**

- In the first paragraph we’ve added details on how we obtained the working dataset (from the women and wealth files), and specified that we are indeed using secondary data.
- We have added details on the calculation of the outcome variables and the covariates.

3. The results section is not telling a story but rather reads like a mere description of tables. The study makes very interesting findings that are masked by the fixation on describing table, the tables must support the story being told to make for an interesting read.

**Response**: We think this comment raises the distinction between Results and Discussion. We prefer to retain our distinction wherein the Results section is indeed a description of tables whereas, in the Discussion, we try to tell an interesting story. We hope this is acceptable to the Reviewer and the Editor.

C. **Comments by Reviewer #2**
1. Adolescent pregnancy is a very important aspect of reproductive health and this manuscript is a bold attempt to answer some pertinent questions in a country like Cameroon where studies are lacking. The effort to distinguish premarital from marital pregnancy provides more insight and data that can fine tune policies on adolescent pregnancy. There manuscript is well written but there are some areas that will need revision.

**Response:** Thanks!

2. The authors carried out secondary data analysis of DHS survey data over a period of time but they did not discuss how the survey was carried out and provided very little information that can enable the assessment of the quality of the data. More details on these will be needed. Furthermore, even if detailed ethical approval was not required for the study, the authors need to discuss the ethical considerations around using the secondary data.

**Response**

- In the first paragraph of the methods section, we have added an additional sentence on the DHS program.
- The methodology of DHSs is familiar and details of the Cameroon surveys can be found in the main published reports of each of the five rounds. Though the quality of DHS data is acknowledged to be high, they are, of course, far from perfect, as has been documented in many methodological reports. Of particular concern for the purposes of this paper is possible omission of recent births. Our analysis of trends in teenage pregnancy suggests somewhat poor quality of reporting in 2018. With this exception, our results are sufficiently coherent and convincing as to allay doubts about data quality.
- To our knowledge, there are no major ethical considerations around using the DHS data.

3. Lines 121-122, 234-235, 257 and 275 need to be revised.

**Response**

- Lines 121-122: We have rephrased the beginning of sentence.
- Line 257: We have removed a hanging letter ‘’t’’.
- Line 275: We mistakenly have a period, where we needed a coma.
- Lines 234-235: We did not find anything that’d need to be revised.

4. In the results section, results that relate to the tables should be presented before moving to the figures. The authors tend to mix both, making the presentation of results incoherent in some instances.

**Response**: We have revised the beginning of the 2^nd^ sentence of the results section to ensure clarity from Table 1 and Figure 1.

5. What do they author mean by the speed of entry into marriage after pre-marital pregnancy? I understand this was derived from the time difference but care should be taken when using such descriptions. It is better to use hazard of entry consistently.

**Response**: We have replaced speed with hazard.

6. Authors should avoid using prevalence. They should use proportions consistently.

**Response**: We have replaced ‘’prevalence of premarital pregnancy” with “likelihood of premarital agency”.

7. The relationship between premarital pregnancy and education was not clear. In one section the author mention that the prevalence of premarital pregnancy increases with education level and in another section they state that: “intermediate levels of schooling (primary or lower secondary) recorded consistently higher probabilities of having a premarital pregnancy than those with no schooling or with the highest level of schooling”. This should be clearly articulated in the text. In the discussion section, enough explanation was not given for this.

**Response**: We have expanded the text in Line 168 to clarify further.

8. In general, for the discussion section, the authors have not used enough contemporary literature that will be relevant to the topic under consideration.

**Response**: We have added two additional contemporary references (now #35 and #36).

In **conclusion**, we believe we have adequately addressed all concerns and comments raised by the Editor and Reviewers.
